# Effect of heat inactivation for the detection of severe acute respiratory syndrome-corona virus-2 (SARS-CoV-2) with reverse transcription real time polymerase chain reaction (rRT-PCR): evidence from Ethiopian study

**DOI:** 10.1186/s12879-022-07134-7

**Published:** 2022-02-21

**Authors:** Belete Woldesemayat, Gebremedihin Gebremicael, Kidist Zealiyas, Amelework Yilma, Sisay Adane, Mengistu Yimer, Gadissa Gutema, Altaye Feleke, Kassu Desta

**Affiliations:** 1 HIV/AIDS Disease Research Team, TB and HIV/AIDS Disease Research Directorate, Ethiopian Public Health Institute, P.O. Box 1242, Addis Ababa, Ethiopia; 2 Department of Medical Laboratory Sciences, College of Health Sciences, Addis Ababa University, Addis Ababa, Ethiopia

**Keywords:** COVID-19, Ct value, Heat inactivation, rRT-PCR, SARS-CoV-2

## Abstract

**Background:**

Coronavirus disease 2019 (COVID-19) has been a major public health importance and its specimen needs to be handled safely due to concerns of potential transmissibility to health care workers. Heat inactivation of the sample before nucleic acid isolation might permit safe testing processes. Hence, it is important to assess the effect of heat inactivation on SARS-CoV-2 RT-PCR detection in resource limited settings.

**Methods:**

An experimental study was conducted at Ethiopian Public Health Institute (EPHI) from September 25 to October 15, 2020. A total of 188 Oro-pharyngeal swabs were collected from COVID-19 suspected cases, referred to EPHI for SARS COV-2 testing. One batch of the sample was inactivated at 56 °C heat for 30 min, and the other batch was stored at 4 °C for a similar period of time. RNA extraction and detection were done by DAAN Gene kit protocols. Abbott m2000 RT-PCR was used for amplification and detection. Data analysis was done by using SPSS version 23.0; Chi-square and Pearson correlation test for qualitative and semi-quantitative analysis were used. p-value < 0.05 was considered as statistically significant.

**Results:**

Out of 188 total samples, 119 (63.3%) were positive and 69 (36.7%) were negative in the non-inactivated group. While, 115 (61.2%) of samples were positive and 73 (38.8) were negative in heat inactivated sample batch. Rate of positivity between groups did not have statistically significant difference (p > 0.05). The mean Cycle threshold (Ct) value difference between the two groups of ORF1a/b gene and N gene was 0.042 (95% CI − 0.247–0.331; t = 0.28; p = 0.774) and 0.38 (95% CI 0.097–0.682; t = 2.638; p = 0.010) respectively**.**

**Conclusion:**

Heat inactivation at 56 °C for 30 min did not affect the qualitative rRT-PCR detection of SARS-CoV-2. However, the finding showed that there was statistically significant Ct value increment after heat inactivation compared to untreated samples. Therefore, false-negative results for high Ct value (Ct > 35) samples were found to be the challenge of this protocol. Hence alternative inactivation methods should be investigated and further studies should be considered.

## Background

Coronavirus diseases 2019 (COVID-19) was announced as a global pandemic on March 11, 2020, by World Health Organization (WHO) [1]. As of December 12/2021, severe acute respiratory syndrome-2 (SARS CoV-2) infections have been confirmed in nearly 269 million people, and about 5.3 million deaths reported globally [2]. Laboratory testing for COVID-19 has a major importance to show a real scenario of the detected cases in this pandemic era. The testing method can be Nucleic Acid Amplification Test (NAAT) or Serological tests [3]. However, the new recommended point-of-care serological test was failed to be recommended by WHO to use in any setting, including in clinical decision-making rather than in research settings due to window time [4]. Therefore, detection of SARS-CoV-2 RNA with molecular methods (either PCR-based method or deep sequencing) is the gold standard method for the diagnosis of COVID-19. Reverse transcription real-time polymerase chain reaction (rRT-PCR) are using specific sequences of genes that encode the RNA dependent RNA polymerase (RdRP), nucleocapsid (N), envelope (E), and spike (S) proteins of the virus [5]. However, currently available diagnostic method of COVID-19 either PCR-based or deep sequencing have limitations in terms of reliability, accuracy, and accessibility of testing [6]. Because, these detection methods are dependent on the presence of viral genome in sufficient amounts that can be amplified [7]. In addition, in the process of the analytical procedure, there are a lot of challenges including sample collection, transportation, treatment, extraction procedure, amplification and detection [6]. Moreover, according to WHO biosafety guideline COVID-19 molecular testing by using clinical specimens should be performed at least in Biosafety Level 2 (BSL-2) laboratory [8]. Therefore, initial sample processing (before inactivation) of all specimens should be performed in a properly maintained and calibrated biosafety cabinet (BSC). In some condition, including in point of care testing, specimen handling and testing out of designated testing centers, conducting different field surveys settings and when performing automation extraction might be unable to use BSC’s. Thus, specimen treatment (inactivation of SARS-CoV-2) prior to sample handling or extraction could be essential to protect health care workers from nosocomial transmission of COVID-19 [8–10].

Inactivation of SARS-CoV-2 can be done by different methods; like chemical inactivation using 0.5% of Povidone-Iodine oral antiseptic, and/or 70% alcohol can be rapidly inactivate the virus within 30 s contact time [11]. Lysis buffers which are available in RNA extraction kit are also effective for SARS-CoV-2 inactivation without additional means [8]. However, some detergents using for sample treatment can inhibit PCR reactions. Some of cannot inactivate viruses properly without RNA degradation [6]. Different strain of the virus could be inactivated with related temperature range [12]. The famous method of SARS-CoV-2 inactivation at 56 °C for 30 min prior to extraction procedures was showed that leads to a clear drop of viral infectivity (> 5 Log10 reduction) [10, 13]. The other SARS-CoV-2 inactivation at 60 °C for 60 min, 92 °C for 15 min, 80 °C for 5 min, and 100 °C for one minute also could be resulted significantly decrease the viral infectivity in a clinical specimen [13, 14]. However, sample treatment that use heat before molecular testing might destroy the viral RNA and can lead to false-negative results [15].

Since COVID-19 outbreak occurred in Ethiopia, laboratory testing is conducted with NAAT by using RT-PCR technique from Nasopharyngeal or Oro-pharyngeal specimens with automated or manual extraction and detection methods. In this pandemic time expansion of the testing capacity should be important steps that boost the prevention and control strategy of COVID-19 throughout the country. However, resource limitations including level 2 biosafety cabinets are the major challenge. In addition to this, automated extraction and detection are conducted by using different type of instruments in the absence of biosafety cabinet. In this sense, viral inactivation is a mandatory process in order to protect health care workers from the nosocomial transmission of SARS-CoV-2. Currently, in our context, we are using heat at 56 °C for 30 min for viral inactivation. But some shreds of evidence showed that viral inactivation for RNA viruses, including SARS-CoV-2 lead to a false-negative result. Hence we have aimed to evaluate the effect of heat inactivation in the detection of SARS-CoV-2 by using clinical samples in Ethiopian context and generate baseline information for further study on the use of inactivation method prior to nucleic acid extraction for COVID-19 testing that could be applicable in similar settings.

## Methods

This experimental study was conducted at EPHI, National HIV Reference laboratory from September 25 to October 15, 2020. A total of 188 Oro-pharyngeal swabs were collected from COVID-19 suspected cases and referred to EPHI for SARS COV-2 testing during the study period. Oro-pharyngeal sample was collected by trained sample collectors, with 3 ml viral transport media (VTM) (Miraclean technology, Shenzhen, China).Specimens were transported to EPHI, HIV reference laboratory by triple packaging system and the tertiary containers were opened inside the biosafety cabinet level 2 (BSCL-2).

From each, Oro-pharyngeal (throat swab) sample, 500 µl sample was aliquoted into two sterilized cryogenic tubes. Then, one batch of the sample was inactivated at 56 °C heat for 30 min in a water bath and the other batch of the sample was stored at 4 °C for a similar period of time. Both batches of the sample were tested with a lot of 20 samples and 2 controls (1 positive and1 negative), were included throughout the procedure (in extraction and detection) except the last batch of samples, which contained 8 samples and controls. During extraction and master mixing, every step was performed based on standard operating procedure (SOP), every batch of testing was treated similarly, except one group inactivated with heat and the other is not, to minimize factors that can affect the result. The RNA extraction was done by DAAN Gene spin column-based manual extraction kit, manufactured by DAAN Gene Co., Ltd of Sun Yat-sen University, Guangzhou China. In this method of nucleic acid isolation/purification 0.2 ml of VTM throat swab sample was used for manual extraction. In short, 0.2 ml of samples was mixed with 50 µl of proteinase K and 200 µl of lysis buffer. The lysed samples were heated for 10 min on a dry heat block at 72 °C. Extracted nucleic acid was precipitated by using 250 µl of absolute ethanol and after subsequent washing steps adds 50 µl of elution buffer. Finally, approximately 50 µl of eluate was collected with 1.5 ml eppendrof tube. The amplification/detection method of this kit is based on one-step rRT-PCR technique. In this method, ORF1a/b and N genes are selected as the conserved region of DAAN technology for amplification and detection of target regions. Specific primers and fluorescent probes are designed (N gene probe is labeled with Carboxyfluorescein (FAM) and ORF1a/b probe with VIC) for the detection of 2019 novel Coronavirus RNA in the specimens. The final master mix preparation was 5 µl of eluate was added to 20 µl of master mix for a volume of 25 µl. rRT-PCR was performed on the Abbott m2000 RT-PCR (open mode). Based on the manufacturer instruction cycling conditions was as follows: hold for 15 min’s at 50 °C, hold for 15 min’s at 95 °C, then 45 cycles of 94 °C for 15 s, and 55 °C for 45 s.

The result interpretation was done based on the manufacturer recommendation, Ct value > 40 and no amplification curve in the FAM and VIC channel, but amplification curve in Cy5 channel was considered as negative (no detectable SARS-CoV-2 RNA in the sample). On the other hand, if the sample had a clear amplification curve in the FAM and VIC channels and Ct value ≤ 40, the sample was considered as positive for SARS-CoV-2 RNA. If the Ct value of ≤ 40 and amplification curve was detected in a single channel of FAM or VIC, and there was no amplification curve in the other channel, the results were re-tested and the repeated result takes as the final result.

### Data processing and analysis

The data entry and analysis was conducted using statistical software SPSS version 23.0. Descriptive statistics, chi-square and correlation analysis was done to compare different Ct values between groups. A paired T-test was used to measure the mean Ct value difference and to analyze the association between heat inactivated and non-heat inactivated samples Ct value using Pearson correlation coefficient analysis. The consistency analysis value p < 0.05 of the Ct values is considered statistically significant.

## Results

In this study, a total of 188 COVID-19 suspected patients were enrolled and a throat swab sample was collected from September 25 to October 15/2020 referred to EPHI for SARS-CoV-2 testing. Of the total, 108 (57.4%) participants were male and the rest 80 (42.6%) were female. The mean age of the study participants was 32.2 ± 13.1 years.

The positive proportion of non-heat inactivated and heat inactivated samples in this study was 119 (63.3%) and 115 (61.2%) respectively (Table 1). Out of the total samples without heat-inactivated at 56 °C for 30 min (untreated group), 117 (62.2%) and 119 (63.3%) were positive for ORF1a/b gene and N gene respectively. While out of the samples with heat-inactivated at 56 °C for 30 min, 111 (59%) were ORF1a/b positive and 115 (61.2%) were N gene positive. Out of the total N gene-positive, 111 were ORF1a/b gene also positive. Only one sample (0.5%) that treated with heat inactivation was turned to completely double target gene negative after re-tested; while five samples (5/188 = 2.65%) were consistently single gene positive after re-tested. 69 (36.7%) of untreated and 73 (38.8%) of heat inactivated samples were negative for both target genes (ORF1a/b and N gene).

**Table 1:** proportion of qualitative RT-PCR results of non-inactivated and heat inactivated samples in COVID-19 testing, Ethiopia, 2020

Types of group	RT-PCR Qualitative result	
	Positive N (%)	Negative N (%)
Non-heat inactivated sample group	119 (63.3)	69 (36.7)
Heat inactivated sample group	115 (61.2)	73 (38.8)

Of the total samples tested in both methods, 7 (3.7%) discordant results were found (Table 2). Out of the discordant samples; 5 (71.4%) Oro-pharyngeal specimen were double gene negative after the heat-inactivated procedure, but either double or single gene-positive in those treated without heat inactivation procedure. Interestingly, out of the discordant samples; 1 (14.3%) sample was N gene-positive after heat-inactivation, but double gene negative in those treated without the heat-inactivated procedure. Those discordant samples having with single or double gene-positive Ct values were greater than 35 (Table 2). Based on the DAAN gene assay result interpretation after Chi-square McNemar test indicated that, the qualitative detection of SARS-CoV-2 (negativity or positivity) between heat-inactivated at 56 °C for 30 min and non-inactivated group result had no statistically significant difference (x^2^ = 1.584; p = 0.208).

**Table 2:** Discordant result list between inactivated and non-inactivated Oro-pharyngeal specimen in COVID-19 testing, Ethiopia, 2020

Sample ID	Heat Inactivated result		Non-heat inactivated result	
	ORF1a/b gene (Ct value)	N gene (Ct value)	ORF1a/b gene (Ct value)	N gene (Ct value)
ES 020	Negative	Negative	Positive (36.71)	Positive (36.22)
ES 073	Negative	Negative	Positive (37.84)	Negative
ES 086	Negative	Negative	Negative	Positive (36.62)
ES 150	Negative	Negative	Positive (38.2)	Negative
ES 171	Negative	Positive (36.61)	Positive (39.81)	Positive (36.93)
ES 067	Negative	Positive (37.28)	Negative	Negative
ES 068	Negative	Negative	Negative	Positive (36.38)

The effects of heat inactivation at 56 °C for 30 min on the SARS-CoV-2 RT-PCR Ct value were assessed. Out of 188 Oro-pharyngeal swab samples, the ORF1a/b gene average Ct value of the inactivation group and non-inactivation group were 25.43 (95% CI 24.369–26.521) and 25.39 (95% CI 24.336–26.438) respectively. Out of the total sample, 32/188 (17.02%) of samples had low ORF1a/b gene RNA copies, which had the Ct values were greater than 30. While 22 (11.7%) N gene Ct values were greater than 30. The mean non-inactivated ORF1a/b and N gene Ct values greater than 30 were 33.37 (95% CI 32.43–34.32) and 32.52 (95% CI 31.65–33.34) respectively. On the other hand, the inactivated ORF1a/b and N gene Ct values greater than 30 were 34.63 (95% CI 33.46–35.85) and 33.52 (95% CI 32.68–34.29) respectively (Table 3).

**Table 3:** Analysis of Ct value difference between heat inactivated and non-inactivated Oro-pharyngeal specimen in COVID-19 testing, Ethiopia, 2020

Variables	Mean (95% CI)	Paired T-test differences			Non-parametric Wilcoxon test
		Mean difference (95% CI)	T-test	p-value	p-value
Inactivated sample ORF1a/b gene Ct	25.4397 (24.369, 26.521)	0.042 (− 0.247, 0.331)	0.288	0.774	0.871
Non-inactivated sample ORF1a/b gene Ct	25.3978 (24.336, 26.438)				
Inactivated sample N gene Ct	24.1585 (22.925, 25.301)	0.389 (0.097, 0.682)	2.638	0.010	0.000
Non-inactivated sample N gene Ct	23.7689 (22.609, 24.863)				
Inactivated sample ORF1a/b gene Ct > 30	34.6269 (33.455, 35.852)	1.262 (0.435, 2.088	3.114	0.004	0.001
Non-inactivated sample ORF1a/b gene Ct > 30	33.3650 (32.427, 34.321)				
Inactivated sample N gene Ct > 30	33.5155 (32.6796, 34.293)	1.00 (0.439, 1.561)	3.709	0.001	0.000
Non-inactivated sample N gene Ct > 30	32.5155 (31.646, 33.3397)				

**Fig. 1:**
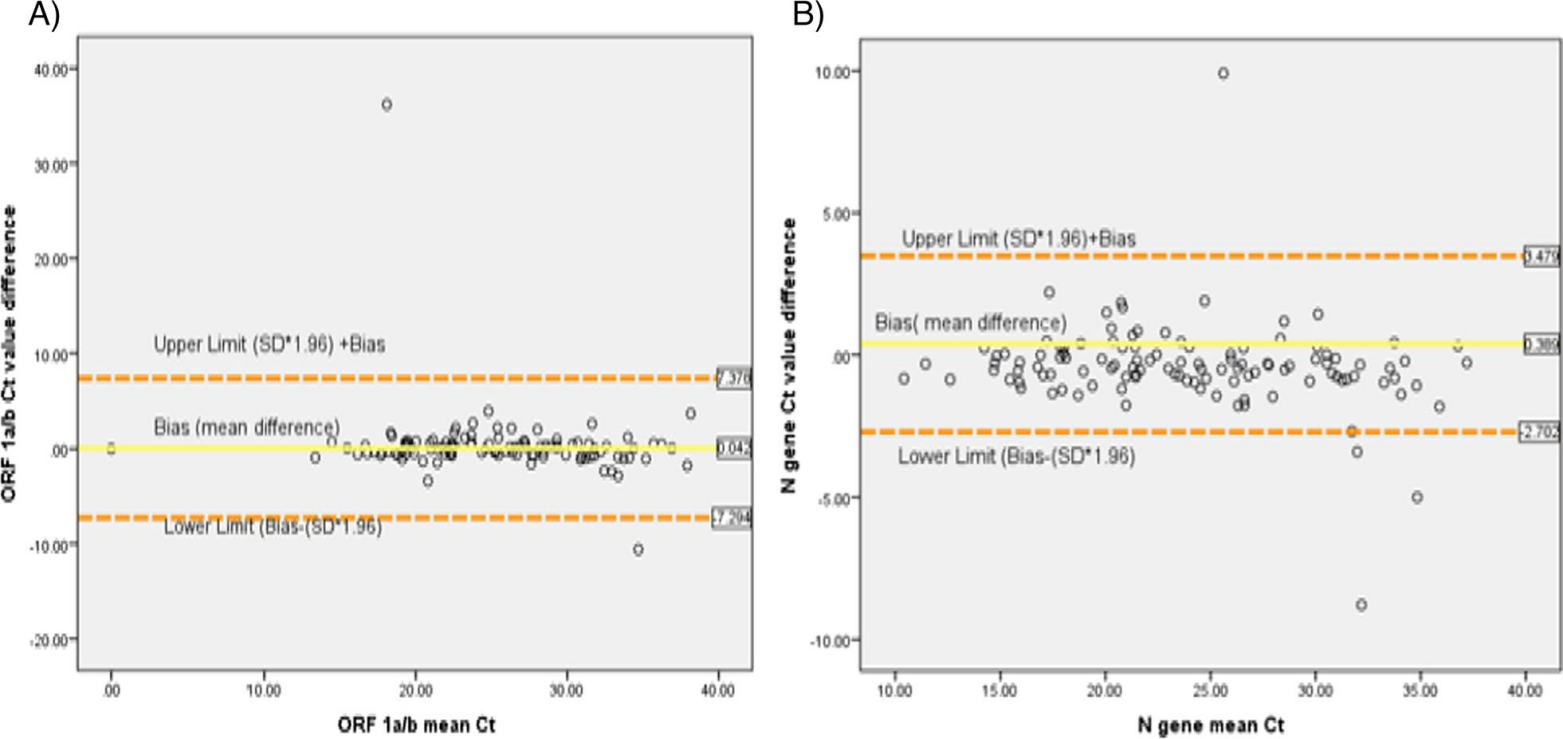
Bland Altman plot of Ct value comparisons between heat-inactivated at 56 °C for 30 min and non-inactivated group in COVID-19 testing, Ethiopia, 2020. **A)** ORF1a/b gene Ct value comparisons between heat treated group and non-inactivated group. **B)** N gene Ct value comparisons between heat treated group and non-inactivated group

As shown in Table 3, there is no significant difference between the ORF1a/b Ct of inactivated and non-inactivated results. The mean difference between the two groups of Ct value was 0.042 (95% CI − 0.247–0.331) and this difference was not statistically significant (t = 0.28; p = 0.774). The Bland Altman comparison showed that, the lower and higher limit of agreement (LOA) between heat treated and non-treated samples were 7.378 and − 7.294 respectively (Fig. 1A). On the other hand, the range of N gene Ct value in both inactivated and non-treated samples was 29.17 (10.83–40.0) and 30 (10.0–40.0) respectively. The mean Ct value for N gene of the heat-treated group was 24.1585 (95% CI 22.925–25.301) and the non-inactivated mean Ct value was 23.768 (95% CI 22.609–24.863). The paired t-test analysis showed that the mean difference between both groups of N gene Ct was 0.38 (95% CI 0.097–0.682). This difference indicated that, inactivated N gene Ct values were significantly higher compared to non-inactivated group (t = 2.638; p = 0.010). The Bland Altman plot also revealed that, inconsistent Ct values were observed below and above the mean difference and the 95% LOA was 3.479, − 2.702 (Fig. 1B). In addition, as indicated in paired T-test analysis, high Ct value (> 30) samples significantly affected with heat inactivation at 56 °C for 30 min. The mean Ct value difference of ORF1a/b and N gene in both inactivated and non-inactivated groups were significantly different (p < 0.05) (Table 3).

**Fig. 2:**
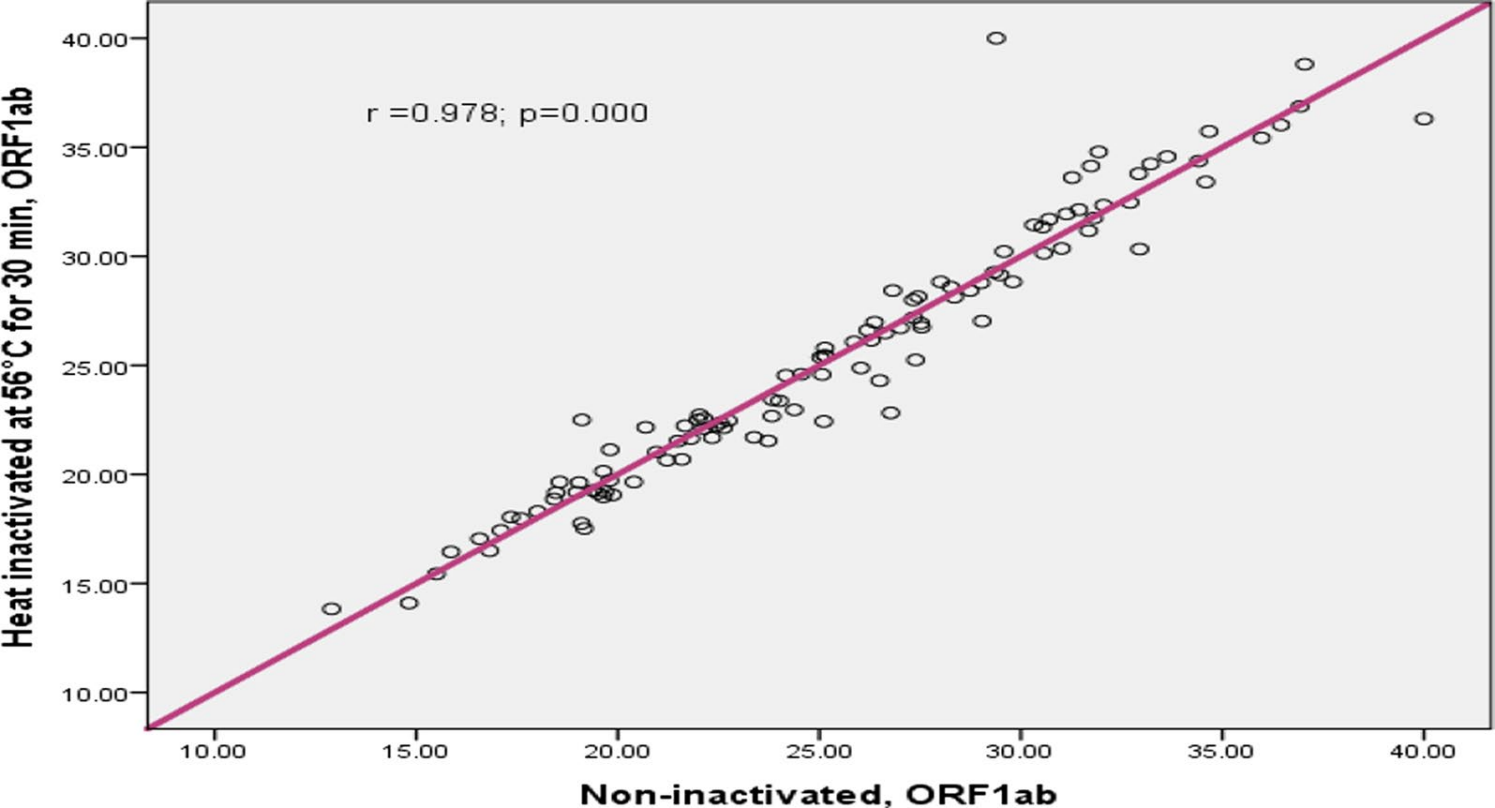
Correlation of inactivated and non-inactivated ORF1a/b gene Ct for Oro-pharyngeal specimen in COVID-19 testing, Ethiopia, 2020

**Fig. 3:**
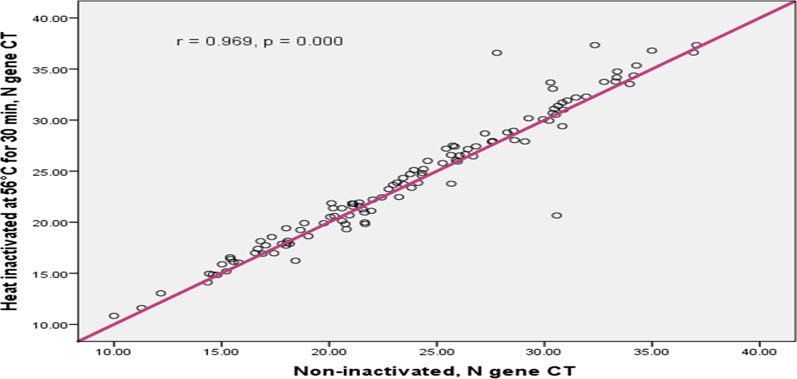
Correlation of inactivated and non-inactivated N gene Ct for Oro-pharyngeal specimen in COVID-19 testing, Ethiopia, 2020

**Fig. 4:**
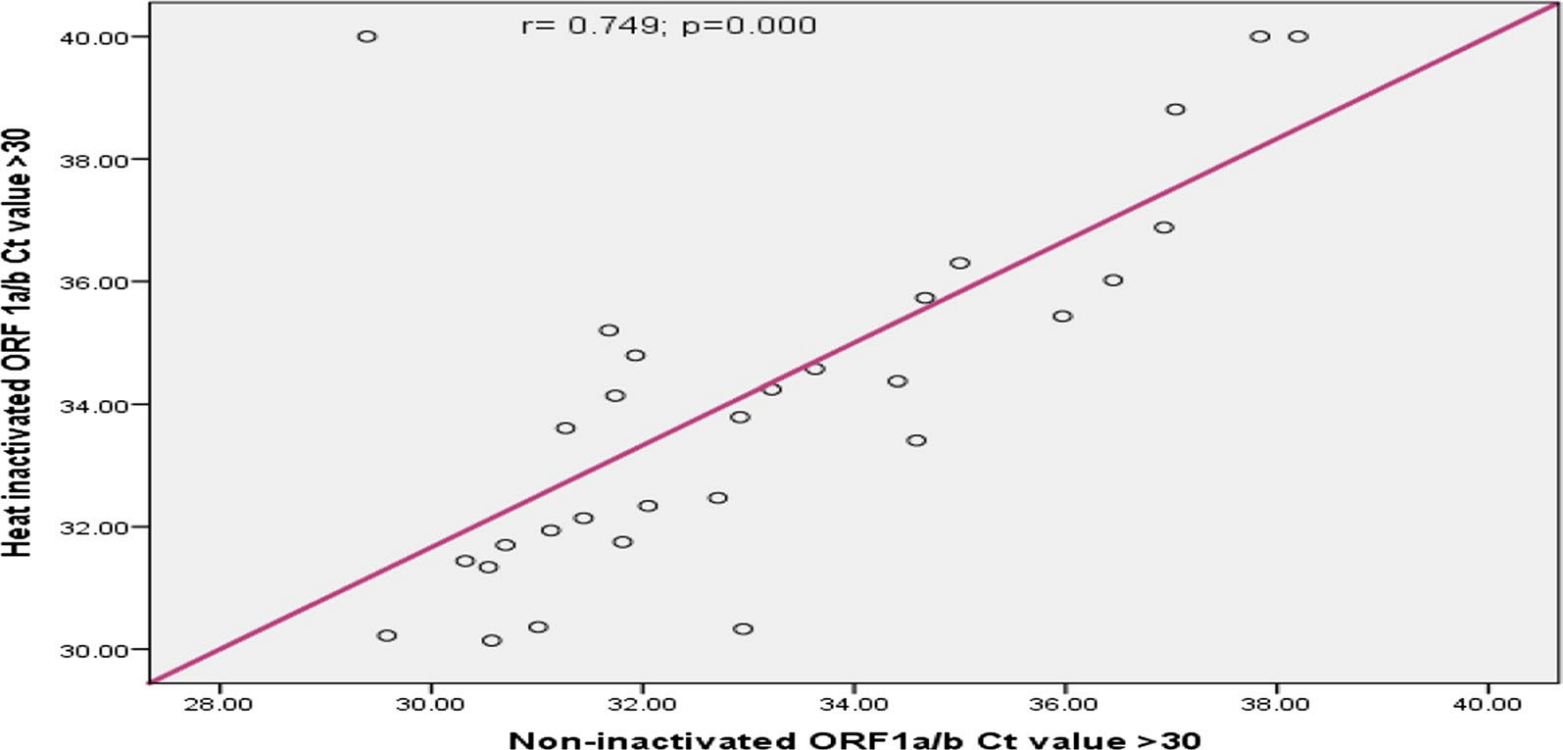
Correlation of inactivated and non-inactivated ORF1a/b gene Ct value greater than 30 for Oro-pharyngeal specimen in COVID-19 testing, Ethiopia, 2020

**Fig. 5:**
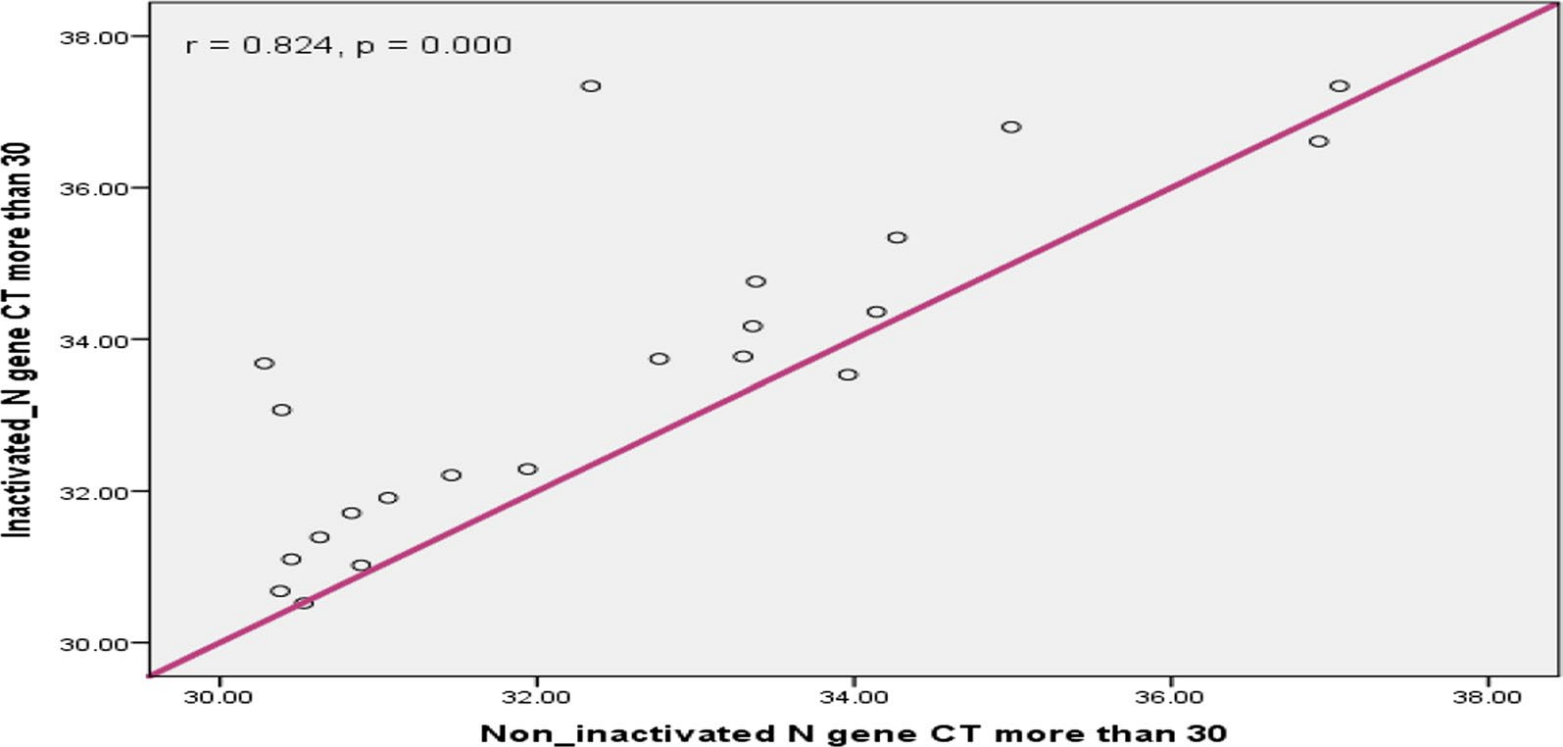
Correlation of inactivated and non-inactivated N gene Ct value greater than 30 for Oro-pharyngeal specimen in COVID-19 testing, Ethiopia, 2020

In this study the concordance between inactivated and non-inactivated sample results was compared and Pearson correlation analysis showed that, the ORF1a/b gene Ct values of inactivated sample and non-inactivated sample had excellent correlation (r = 0.978; p < 0.001); (Fig. 2). Similarly as Fig. 3 shows, the N gene Ct of inactivated and non-inactivated group had excellent correlation (r = 0.969; 95% CI 0 0.934–0.991; p < 0.001). According to Figs. 4 and 5, the ORF1a/b gene Ct value correlation between inactivated and non-inactivated groups had high positive correlation (r = 0.749; 95% CI 0.434–0.935; p = 0.000). The N gene correlation between two groups had also strong positive correlation (r = 0.824; 95% CI 0.634–0.962; p = 0.000).

## Discussion

After COVID-19 declared as a global pandemic, the numbers of suspected cases were increased day today and that needs to maximize laboratory testing capacity by high throughput automated and point of care testing instruments for SARS-CoV-2 detection [6, 9]. For these types of platforms sample inactivation is an important procedure to protect health care workers from the exposure of SARS-CoV-2 infection [9]. However, some studies showed that sample inactivation by using heat can leads to RNA degradation and false-negative results [15, 16]. Therefore, this study was evaluated the effect of heat inactivation at 56 °C for 30 min for SARS-CoV-2 detection. In this study, seven samples (3.7%) had discordant results between those heat-inactivated and without heat-inactivated matched samples. Out of the total discordant samples, 71.4% of samples were double gene negative during the heat-inactivated procedure; but either double or single gene-positive in those without the heat-inactivated procedure. Those discordant samples having with single or double gene-positive were had more than 35 Ct value. A similar study performed in Beijing, China, showed that, the impact of heat inactivation on samples those had high Ct values (33.37–36.89) were high [16]. This indicates heat inactivation may have a negative impact on decreasing viral load of those samples having Ct values greater than 35. Early identification and detection, early prevention, and control are the currently available method to curbing the rapid spread of COVID-19 infection. However, it may have a negative impact on these measures. Although heat inactivation makes safe for health care workers, false-negative individuals in the community may be transmitted to a wide range of people. On the other hand, the positivity and negativity of the original sample and the heat-inactivated sample result in this study was showed that had no statistically significant difference after a chi-square test (p > 0.05). Similar results were obtained from Renmin Hospital of Wuhan University, China [17], Zhujiang Hospital, China [18], and a study conducted in USA on sputum sample [9] has no significant difference between the non-inactivated and inactivated sample at 56 °C for 30 min for the qualitative detection of SARS-CoV-2. On the contrary, a study conducted in Beijing, China [16] and Yongchuan District Center for Disease Control and Prevention of Chongqing, China [19] were indicated that the qualitative detection of SARS-CoV-2 was significantly impacted with heat inactivation at 56 °C for 30 min. This difference might be due to variation in sample size between experiments, type of detection/extraction reagents, sample type and viral strain difference by itself. However, it is difficult to know the impact of this result on the transmission of SARS-CoV-2, as the virus is new, with lots of unknown characteristics and spreading as fast as a forest fire. In this study, the effect of heat inactivation at 56 °C for 30 min on the Ct value of matched samples was analyzed, and the average Ct value difference between non-inactivated and heat-inactivated at 56 °C for 30 min were 0.04 and 0.38 for ORF1a/b gene and N gene respectively. It means that after heat inactivation the ORF1a/b gene and N gene Ct was increased by 0.04 and 0.38 averagely on each sample respectively. Based on paired T-test analysis, the ORF1a/b gene Ct value increment after heat inactivation was not statistically significant (t = 0.28; p > 0.05), whereas the N gene Ct values between inactivated and the non-inactivated group were statistically significant (t = 2.64; p = 0.01). In addition to this, the mean Ct value difference of heat-treated and untreated group sample greater than 30 Ct of ORF1a/b and N gene were 1.26 and 1.00 respectively. These average Ct value increment while heat inactivation was statistically significant in both N and ORF1a/b gene (p < 0.05). Similar studies reported by Pan et al. [16] and Chen et al. [15] both were from China and indicated that heat inactivation prior to extraction can significantly reduce the number of RNAcopies (increased Ct values compared to the original sample). This may result in false-negative during heat inactivation procedure and favour viral transmission. However, the studies conducted in Charite University, Berlin, Germany [13], Renmin Hospital of Wuhan University, Huazhong China [17], Republic of South Korea [20] and Zhejiang University School of Medicine, China [21] revealed that Ct values of heat-inactivated at 56 °C for 30 min group and the non-inactivated group had no statistically significant difference. These difference might be due to sample size, type of sample or and type of strain difference circulating in the community which need additional investigations.

In this experimental study, the correlation between inactivated and non-inactivated samples was also examined. As a result, Pearson correlation analysis was performed and the analysis showed that, the ORF1a/b and N gene Ct values of inactivated sample and non-inactivated sample had excellent correlation (r = 0.978; p < 0.001); and (r = 0.969; p < 0.001) respectively (Figs. 2 and 3). The result reported from the First People’s Hospital of Zhaoqing, Zhao Qing City, China, the Ct value of ORF1a/b gene and N gene were perfectly correlated between inactivated at 56 °C for 30 min and non-inactivated samples [15].

Our study has some limitations; first, we have used only Oro-pharyngeal swabs, so we cannot conclude for another type of samples; like sputum, saliva, blood and stool samples. Second, we didn’t perform SARS-CoV-2 viral quantification to show the exact viral copy difference between inactivated and non-inactivated sample results. And third, we did not perform viral infectivity analysis because of lack of infrastructure.

## Conclusion

In conclusion, our result showed that heat inactivation at 56 °C for 30 min does not have statistically significant effect for the qualitative rRT-PCR detection (positivity or negativity rate of detection) of SARS-CoV-2 infection. However, this study showed that there was statistically significant Ct value increment after heat inactivation at 56 °C for 30 min compared to untreated samples. So, a false negative result in high Ct value (especially greater than 35) might be the challenge of this protocol. Finally for SARS-CoV-2 viral inactivation prior to sample handling or extraction other inactivation methods rather than heat inactivation and further studies should be considered. By replicating this study the real effect of heat inactivation on the detection of SARS-CoV-2 viral genomic materials could be known which will have an impact on the diagnosis and prognosis of COVID-19 patients specifically in resource-limited settings.

## Data Availability

All data generated or analysed during this study are included in this published article. The data that support the findings of this study are available from the corresponding author on reasonable request.

## Abbreviations

BSC: Biological safety cabinet
BSCL2: Biosafety cabinet level 2
CDC: Center for disease control
COVID-19: Coronavirus disease 19
Ct: Cycle threshold
EPHI: Ethiopian Public Health Institute
IC: Internal control
NAAT: Nucleic acid amplification test
ORF1a/b: Open reading frame 1a/b
POC: Point of care
rRT-PCR: Reverse transcriptase real time polymerase chain reaction
SARS CoV-2: Sever Acute Respiratory Syndrome Corona Virus-2
SOP: Standard operation procedure
VTM: Viral transport media
WHO: World Health Organization
